# Supplementary material for: Fin whale singing decreases with increased swimming speed
Source: R Soc Open Sci. 2019 Jun 5;6(6):180525. doi: 10.1098/rsos.180525 (PMC6599786; doi:10.1098/rsos.180525)
Supplement: Table S2 PDF [file rsos180525supp3.pdf]

Variable	N	Mean	St Dev	Median	SEM	Min	Max
Track-length (km)	163	86.2	89.8	51	7.03	10	430
Track-duration (h)	163	14.7	14.7	9.3	--	1.1	70.2
Track total-singing-duration (h)	163	15.8	14.8	10.3	--	1.1	70.4
Number of locations per track	163	8.4	7.1	6	--	3	39
Track song duration (min)	163	9.8	5.6	8.9	0.44	0.7	25.3
Songs per track	163	49.3	41.77	36	--	6	226
Track Swimming Speed (km/h)	163	6.7	3.4	6.2	0.27	1.1	16.8
Min swimming speed (km/h)	163	4.1	3.2	--	--	n/a	13.8
Max swimming speed (km/h)	163	8.7	5	--	--	0.5	n/a
Track Duty-cycle (%)	163	54.3	21.9	53.7	1.7	9.2	95
Min Duty Cycle (%)	163	23.7	30.1	--	--	n/a	93.6
Max Duty Cycle (%)	163	77.3	21	--	--	11	n/a
Segment swimming speed	1208	6.36	3.89	5.69	--	0.1	22.6
Segment duty-cycle (%)	1208	52.5	34.6	64.45	--	0	96.3
Song durations (min)	8040	9.4	6.64	8.9	--	0.3	32.5
Inter-song-interval (min)	7877	9.7	33.72	2.5	--	0.3	1061
