# Supplementary figures and images for: Fin whale singing decreases with increased swimming speed
Source: R Soc Open Sci. 2019 Jun 5;6(6):180525. doi: 10.1098/rsos.180525 (PMC6599786; doi:10.1098/rsos.180525)

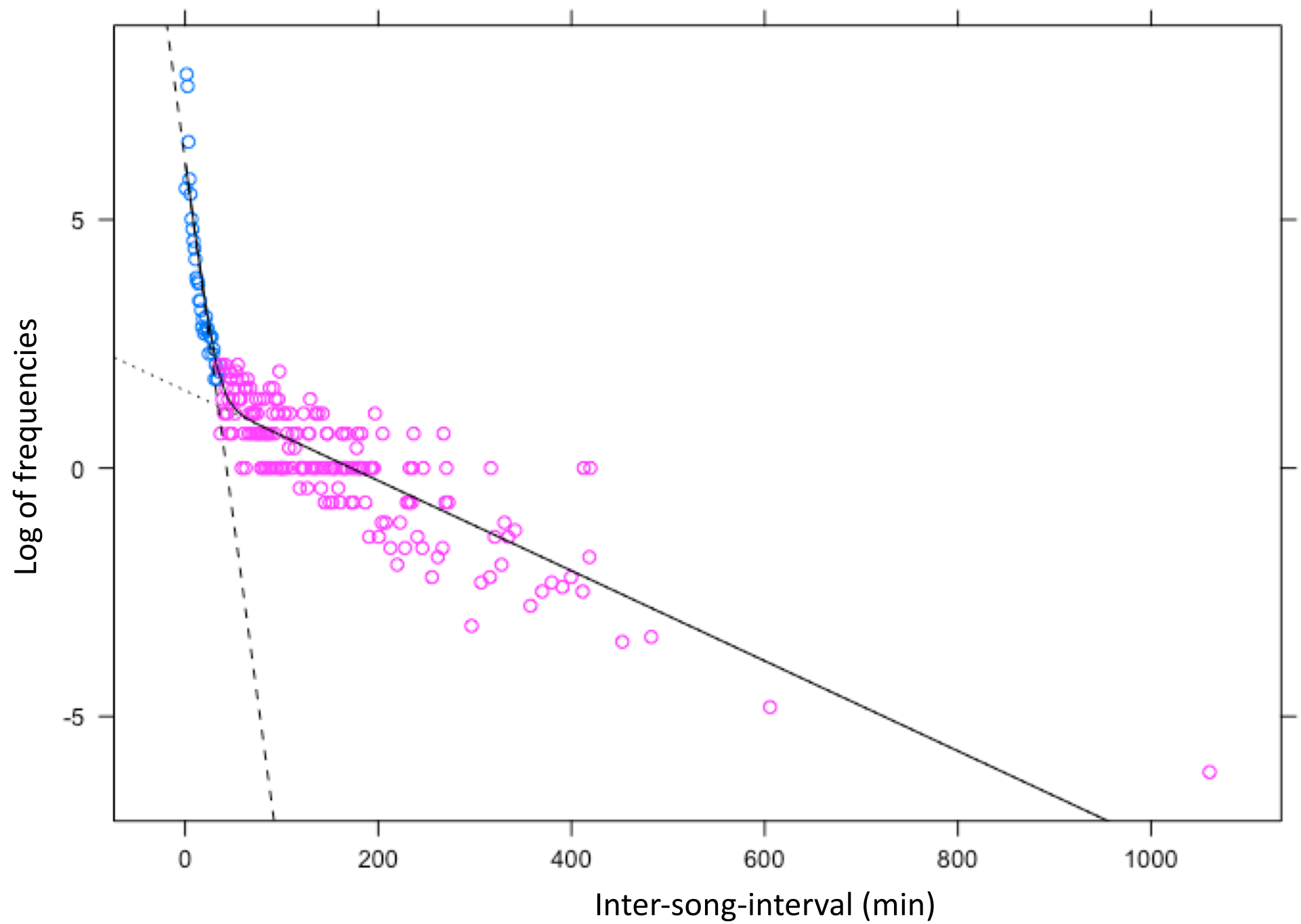

Supplement: Fig_S-3 [file rsos180525supp9.pdf]
